# Food allergy and anaphylaxis – 2061. Clinical symptoms and molecular characterization of hazelnut allergy in Italian children

**DOI:** 10.1186/1939-4551-6-S1-P144

**Published:** 2013-04-23

**Authors:** Marco Albarini, Alessandro Fiocchi, Lamia Dahdah, Gianni Melioli, Oscar Mazzina, Fabrizio Veglia, Antonella Marelli

**Affiliations:** 1 Melloni Paediatria, the Melloni University Hospital, Milano, Italy; 2 Clinical Pathology Laboratories, Department of Experimental and Laboratory Medicine, Istituto G. Gaslini, Genova, Italy; 3 Immunodiagnostics, Thermo Fisher Scientific, Milano, Italy

## Background

Hazelnut allergy is associated with sensitization to PR-10 (Cora1) and profillin (Cora2), but several other allergens in hazelnut, including Cor.a.8 (lipid transfer protein) and Cor.a.9 (11S globulin) may be relevant. We targeted this study to the association among different sensitization profiles and symptoms in pediatric hazelnut allergy.

## Methods

Thirty-five children (median age: 8.3, range2.2–14.2 yr) reported with immediate reactions to hazelnut ingestion underwent food challenge (DBPCFC) between April 2007 and May 2012. Skin prick test (SPT), specific IgE determination at ImmunoCAP with whole food allergens and with allergen components at ImmunoCAP ISAC microarray chip were performed. The molecular profile of reactivity was compared to SPT and specific IgE testing. This report focuses on the hazelnut-associated allergens Cor.a.1.01, Cor.a.1.04, Cor.a.8 and Cor.a.9.

## Results

16 children (10M, 6F) tested positive (group A) and 19 (16M, 3F) negative (group B) at DBPCFC. On a 3-mm wheal diameter cut-off, 100% were SPT-positive in group A, 47.3% in group B. At ImmunoCAP, 100% returned positive to hazelnut in group A, 78.9% in group B (0.35 kUI/L cut-off). Components detected at ISAC were evaluated in the context of the relevant families: PR-10, LTP and 11S globulins. 56.2% were Cor.a.1.1010-positive in group A, 26.3% in group B; 43.7% were Cor.a.1.0401-positive in group A, 15.7% in group B; 12.5% were Cor a 8 positive in group A, 10.5% in group B; only one child returned Cor a 9 positive in group A, none in group B.

## Conclusions

A strong sensitization to PR-10 was found in hazelnut-allergic children. Compared to the existing caseloads, our proportion of sensitization to Cor a 1 confirms a sensitization profile different from adults. Cor.a.8 LTP, described as a marker of severe hazelnut allergy in Mediterranean populations, does not play a major role in our group. Cor a 9 sensitization is exceptional. The association among symptoms and sIgE profile should be carefully investigated considering not only the natural history of the patient's sensitization, but also the clear differences between IgE mediated sensitization and clinically evident food allergy.

